# Cultivation and Differentiation Change Nuclear Localization of Chromosome Centromeres in Human Mesenchymal Stem Cells

**DOI:** 10.1371/journal.pone.0118350

**Published:** 2015-03-16

**Authors:** Yana I. Voldgorn, Elmira P. Adilgereeva, Evgeny D. Nekrasov, Alexander V. Lavrov

**Affiliations:** 1 Federal State Budgetary Institution «Research Centre for Medical Genetics» of the Russian Academy of Medical Sciences, Russia, 115478, Moscow, Moskvorechie, 1; 2 State Budgetary Educational Institution of Higher Professional Education “Russian National Research Medical University named after N.I. Pirogov” of Ministry of Health of the Russian Federation, Russia, 117997, Moscow, Ostrovityanova str., 1; 3 Moscow Institute of Physics and Technology (State University), Russia, 141700, Moscow Region, Dolgoprudny, Institutskiy per., 9; Pohang University of Science and Technology (POSTECH), REPUBLIC OF KOREA

## Abstract

Chromosome arrangement in the interphase nucleus is not accidental. Strong evidences support that nuclear localization is an important mechanism of epigenetic regulation of gene expression. The purpose of this research was to identify differences in the localization of centromeres of chromosomes 6, 12, 18 and X in human mesenchymal stem cells depending on differentiation and cultivating time. We analyzed centromere positions in more than 4000 nuclei in 19 mesenchymal stem cell cultures before and after prolonged cultivation and after differentiation into osteogenic and adipogenic directions. We found a centromere reposition of HSAX at late passages and after differentiation in osteogenic direction as well as of HSA12 and HSA18 after adipogenic differentiation. The observed changes of the nuclear structure are new nuclear characteristics of the studied cells which may reflect regulatory changes of gene expression during the studied processes.

## Introduction

Nuclear location (chromosomal territory, CT) is an important characteristic of each chromosome. The term "chromosome territory" now-a-days refers to that part of the nucleus, in which hybridization with labeled DNA reveals material of a single chromosome. Part of the nucleus in which chromatin could not be detected using standard methods of microscopy is called interchromatin domain. This description is not ideal, since in reality the material of the chromosome may be located outside its territory and even inside the territory of the other chromosome, and interchromatin space may contain chromatin loops [1].

Up to date, reasons and mechanisms of CT localization in the certain parts of the nucleus remain unclear. However it was previously shown that CT position depends on several factors including cell cycle phase, cell type [2] and differentiation process [3–5]. These facts were revealed in erythroid differentiation [6], adipogenesis [4], T-lymphocyte maturing [7] and spermatogenesis [8]. The aspects of CT movements during differentiation are enlightened in Bartova and Kozubek review [9]. It was also shown that analysis of co-localization of several chromosomes reveals stable CT-association patterns in cells from different tissues [2]. Such processes as DNA damage also might induce a large-scale spatial relocalization of CT [10]. Thus, CT position and structure differ in different types of cells and tissues, however some stable patterns can be found among them.

Altered chromosome positioning may lead to mutations and nuclear dysfunctions and result in diseases including cancer. Larger chromosomes have a higher frequency of interchromosomal rearrangements. However for smaller chromosomes, such as gene-dense human chromosomes 17, 19, and 22 the frequency of observed translocations depends rather on the nuclear position than on the size of the chromosome. Active chromosomes in the nuclear center (such as chromosome 19) seem to undergo genomic changes at higher rates than that of inactive chromosomes at the nuclear periphery (such as chromosome 18) [11].

Study of CT in undifferentiated cells such as MSC is of particular interest. MSCs are able to differentiate into mesenchymal cells such as osteocytes, chondrocytes, adipocytes, as well as in some non-mesenchymal cell [12–17]. Their plasticity made them an ideal cell source for regenerative medicine and cell therapy. MSCs have good proliferation potential and are often cultivated before transplantation and/or differentiation into targeted cells depending on the clinical application. MSC cultivation allows generating millions of cells in several days however long cultivation results in culture ageing and spontaneous differentiation which should be avoided or at least controlled in clinical applications. In some studies it’s also argued that MSC may induce tumor growth [18] what is also might be affected by chromosome repositioning during cultivation and ageing. So MSCs are a good model to investigate the basic role of chromosome organization for cell differentiation on one hand and other hand are thought to be an important material for clinical use which is not yet thoroughly characterized.

The purpose of this study was to identify differences in the location of centromeres of chromosomes 6, 12, 18 and X in the MSC depending on differentiation and cultivating time.

## Material and Methods

### Cell isolation, cultivation and fixation

MSCs were obtained from adipose tissue of 19 healthy donors. The donors gave written informed consents and the work was approved by the Ethic Committee of Federal State Budgetary Institution «Research Centre for Medical Genetics» of the Russian Academy of Medical Sciences, protocol #6 from 12/07/2012. Cell cultures were prepared by standard methods for the preparation of cells for clinical transplantation. Cells were cultured in complete growth medium (CGM): DMEM/F12, 2 mM L-glutamine, 10% FBS, 8U/ml heparin, 100U/ml penicillin, streptomycin 100μg/ml. Cells were passaged every 3–4 days. Passages 1–4 were attributed to early passages, while after 7 passages they were considered as late. The cells were seeded on glass before fixing. Cells were fixed in ice-cold 100% methanol for 8 minutes, washed with phosphate buffer and distilled water and dehydrated in 70%, 85% and 96% ethanol for three minutes each.

Human lymphocytes were obtained from 11 healthy donors. Lymphocytes were grown in RPMI 1640 medium at 37°C, incubated with Versen for 1 minute, then 0.25% trypsin was added. Cells were harvested by centrifugation and incubated in 0,075mM KCl at 37°C for 9 minutes. Then the cells were fixed with standard fixation procedure by three times incubating in ice cold 3:1 methanol and acetic acid solution for 10 minutes each.

### Cell differentiation

For adipogenic differentiation MSCs were cultivated for 20 days in CGM with 100 μM indometacin, 1 mM dexamethasone, 0,5 mM 1-methyl-3-isobutyl xanthine, and insulin 10 μg/ml. To confirm differentiation fixed cells were stained with Oil Red O (3mg/ml Oil Red O in 60% isopropanol solution) for 15 minutes.

For osteogenic differentiation MSC were cultivated for 17 days in CGM with 50 μM L-ascorbic acid phosphate, 10 mM beta-glycerophosphate and 100 nM dexamethasone. To confirm differentiation fixed cells were stained with Alizarin Red (2% Alizarin Red in 0,1M NH4OH; pH = 5,4) for 15 minutes.

### FISH-analysis and microscopy

The following Vysis probes were used for interphase FISH-analysis: CEP6 (D6Z1), CEP12 (D12Z3), CEP18 (D18Z1), Sergio (DXZ1) alpha satellite DNA for chromosomes 6, 12, 18, X. Preparation and hybridization of the slides were performed according to manufacturer's recommendations.

DAPI was used for nuclear staining. FISH analysis was performed with AxioImager (Carl Zeiss) microscope using 100x immersion objectives. 3D-FISH analysis was performed using confocal LSM-710-NLO microscope (Carl Zeiss). Z-slices were done every 0.346–0.5 mkm.

### Nuclear characteristics

**Fig 1:**
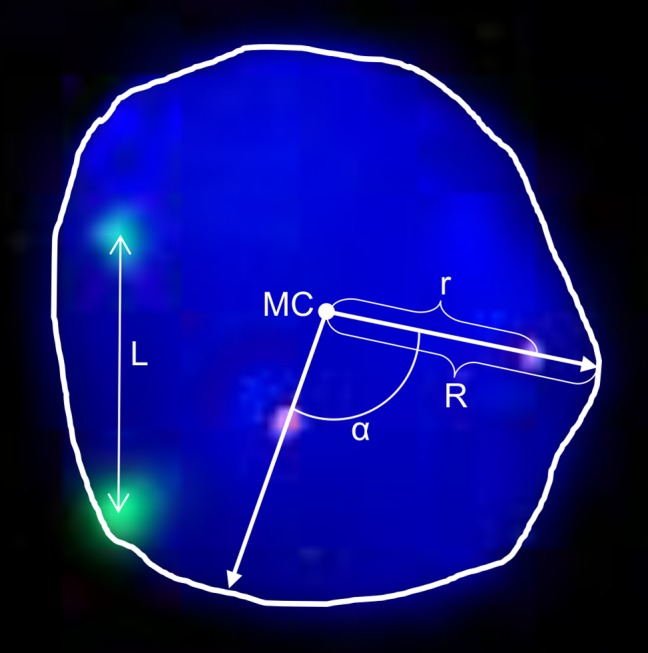
Nuclear characteristics. The nucleus is counterstained with DAPI. The centromere signals are seen as red and green dots. The following characteristics of the nuclear are shown: r is the distance from the nuclear mass center (MC) to the center of the signal, R is radius passing through the center of the signal to the nuclear edge; L is the distance between centromeres; α—is the angle between the signals of two centromeres. We determined relative distance of the centromere (RDC) as r/R for each signal. The distance (L) between all pairs of signals and angels (alfa) were also measured.

2D analysis. Centromere position was determined using a specially developed program Nuclear analyzer. The program defines the center of the nucleus as a mass center and calculates the radial distances of centromere (RDC) using formula RDC = r/R, where r is the distance from the nuclear center to the mass center of the signal, R is radius passing through the center of the signal. All the nuclei were checked manually for the correctness of finding nucleus border and signals inside the nucleus. In addition, the program determines the distance and angle between two signals (Fig. 1). The distance is normalized to the nuclear size by division of the distance in pixels by the square of the nucleus in pixels.

3D analysis was performed by manual measurements of RDC using Zen 2010 software.

### Statistical analysis

Medians and averages are calculated in order to compare our data with other published data. Standard errors (SE) are used with means where applicable unless other stated. However, the distribution of radial distances deviates from normal distributions (according to Lilliefors test and Kolmogorov-Smirnov criteria of normality) and it is incorrect to use parametric statistics. Instead of this we divided nuclear area into five concentric zones at the intervals of 0–0,2; 0,2–0,4; 0,4–0,6; 0,6–0,8 and 0,8–1,0 and analyzed the distribution of RDC in these intervals.

In each culture we analyzed at least 50 randomly selected cells with no aneuploidy of studied chromosomes. Significance of the results was confirmed by statistical analysis using χ2 and Mann-Whitney U-test, Lilliefors test, Kruskal-Wallis test and Kolmogorov-Smirnov test.

## Results

### Nuclear positions of HSA6, HSA12, HSA18 and HSAX

We analyzed more than 4,000 nuclei from 19 different MSC cultures before and after cultivation and differentiation in osteogenic and adipogenic directions.

MSC nuclei are flattened ellipsoids with a relatively small z-size. In flat nucleus centromere signal dimensions are often comparable with the thickness of the nucleus [19], which obstructs adequate z-coordinate detection in 3D FISH-analysis. It was previously shown that PFA fixation does not induce significant changes in 3D structure of the nucleus [20–21] and at the same time MSC nuclei being comparable with fibroblasts retain their 3D characteristics after both PFA and methanol fixation [19]. It was also demonstrated in several studies that 2D-distribution of hybridization signals in classical spread nuclei (after methanol fixation) adequately reflects the position of chromosome territories in the relatively flat and elongated nuclei of fibroblast-like cells [19, 22–23]. We also performed 3D-analysis of 10 cell cultures including cultures at early and late passages, adipogenic and osteogenic differentiated cultures (more than 450 nuclei in total) and found no significant differences in radial distances of the studied chromosomes compared to 2D data. Consequently 2D analysis gives comparable results with 3D analysis in this type of flattened cells but takes less time to scan the slides and doesn’t need sophisticated analysis, so we used 2D microscopy in this study.

We selected for the analysis chromosome 18 (HSA18—gene-poor, located at the nuclear periphery) as one of the most widely used and well-studied chromosomes in this type of studies, chromosomes 6 and 12 (HSA6 and HSA12), which both contain a number of genes associated with undifferentiated status of stem cells (BMP6, HDAC2, RUNX2, OCT4, and NANOG, STELLA, GDF3, respectively), and chromosome X (HSAX), since one of the homologues of this chromosome (Barr body) is usually inactivated in female nuclei and should be placed at the periphery compared to an active one. This difference in positions, if detected may serve as an internal control of our method. It is also unclear whether HSAX is localized in male nuclei in the same way as two female homologues in female. We analyzed the position of centromeres of these chromosomes as centromere localization is proved to correlate with whole chromosome localization in both normal and aberrant cells [24, 25]. However it should be noted that FISH signals might not always represent the whole-chromosome territory and our results relate to the studied centromeres only. The mean and the median were calculated for centromere of each chromosome (Table 1). The medians were 65%, 59%, 46% and 69% for HSA6, HSA12, HSA18 and HSAX centromeres, respectively. RDCs were calculated for each studied chromosome.

**Table 1:** Statistical characteristics of RDCs of the HSA6, HSA12, HSA18 and HSAX.

RDC	Median, %	Mean, %	SD, %	SE, %
HSA6	65	63	21	1
HSA12	59	59	23	1
HSA18	46	48	21	1
HSAX (female)	69	67	21	1
HSAX (male)	70	67	21	2

### Nonrandom position of chromosome centromeres

**Fig 2:**
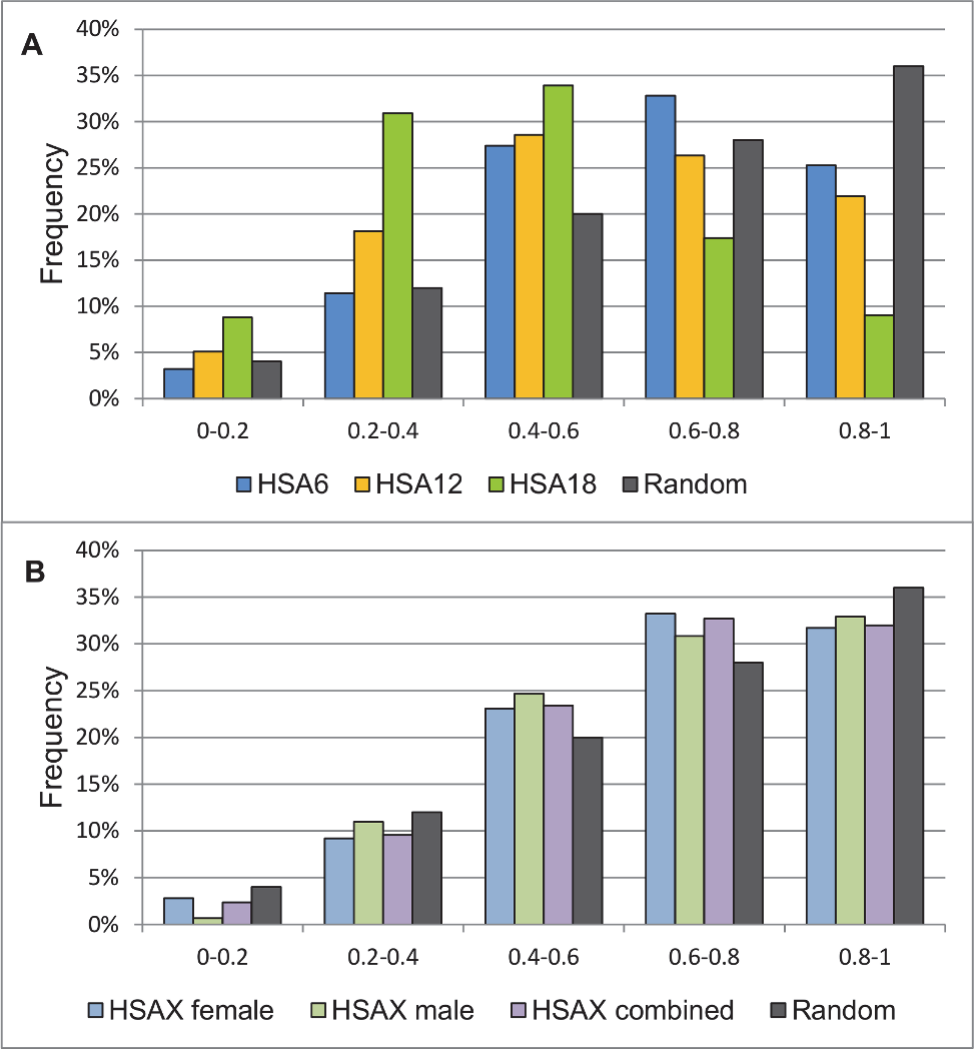
RDCs of HSA6, HSA12, HSA18 and HSAX, and the random signal distribution in the volume of the nucleus. RDCs of HSA6, HSA12 and HSA18 (A) significantly differ from random distribution (χ2 criterion, p = 10–9, p = 10–12 and p = 10–73 for HSA6, HSA12 and HSA18, respectively). For RDCs of HSAX in female and male cultures (B) the difference between random and non-random distribution was not significant (p = 0,09 and p = 0,3 for HSAX in female and male cells, respectively), however combined data from female and male cells gave statistical power to fix the expected differences (p = 0.03).

First we explored that centromeres of the studied chromosomes are not randomly localized in the nucleus. If these signals have been randomly distributed the frequency of their occurrence would increase from the center to the periphery linearly, because the squares of concentric zones—shells (with borders at the following radius: 0–0.2; 0.2–0.4; 0.4–0.6; 0.6–0.8; 0.8–1) grow linearly: 0.13, 0.38, 0.63, 0.88, 1.13. RDCs frequency distributions of HSA6, HSA12 HSA18 significantly differ from the random distribution (p = 10^–9^, p = 10^–12^ and p = 10^–73^ for HSA6, HSA12 and HSA18 centromeres, respectively) (Fig. 2A). The only exception was HSAX (both in male and female cultures) where the difference between random and experimental distribution of the centromere was not significant (p = 0,09 and p = 0,3 for HSAX in female and male cells, respectively) (Fig. 2B). We combined data from HSAX from both female and male cells (there is now difference between them, p = 0.57) which allowed us to detect the expected difference from random distribution (p = 0.03).

### Cultivation of MSC changes position of HSAX centromere

**Fig 3:**
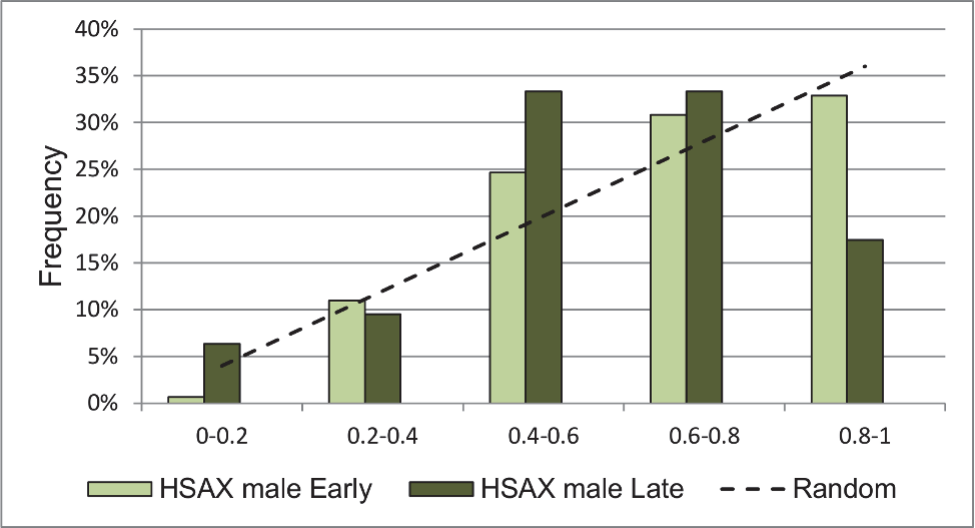
RDCs of HSAX in male cells at early and late passages. RDCs of HSAX in male cells change after prolonged cultivation (p = 0.03). Dashed line demonstrates random distribution for comparison.

We analyzed changes in the RDCs before and after long-time cultivation. The RDCs of HSAX in male cells are different at early and late passages (p = 0,03). At early passages the main quantity of the signal (about 33%) is in the 0,8–1,0 interval. At late passages the frequency of the signal in the region of 0–0,2 is significantly increased. The frequency of finding the centromere in the interval of 0,4–0,8 also increased due to the decrease in the 0,8–1,0 interval (Fig. 3). The position of HSAX centromere in female cells seems to be unchanged at early and late passages, but the distance between homologues decreases at late passages, as compared with early ones (0,0035±0,00014 and 0,0030±0,00014, respectively, p = 0,006, Mann-Whitney Test). It’s interesting to note that there are differences at late passages between RDC distribution of HSAX in male and female cells (p = 0.007). We found no changes for centromeres of HSA6, HSA12 and HSA18 at early and late passages.

### Adipogenic differentiation changes positions of HSA12 and HSA18 centromeres

**Fig 4:**
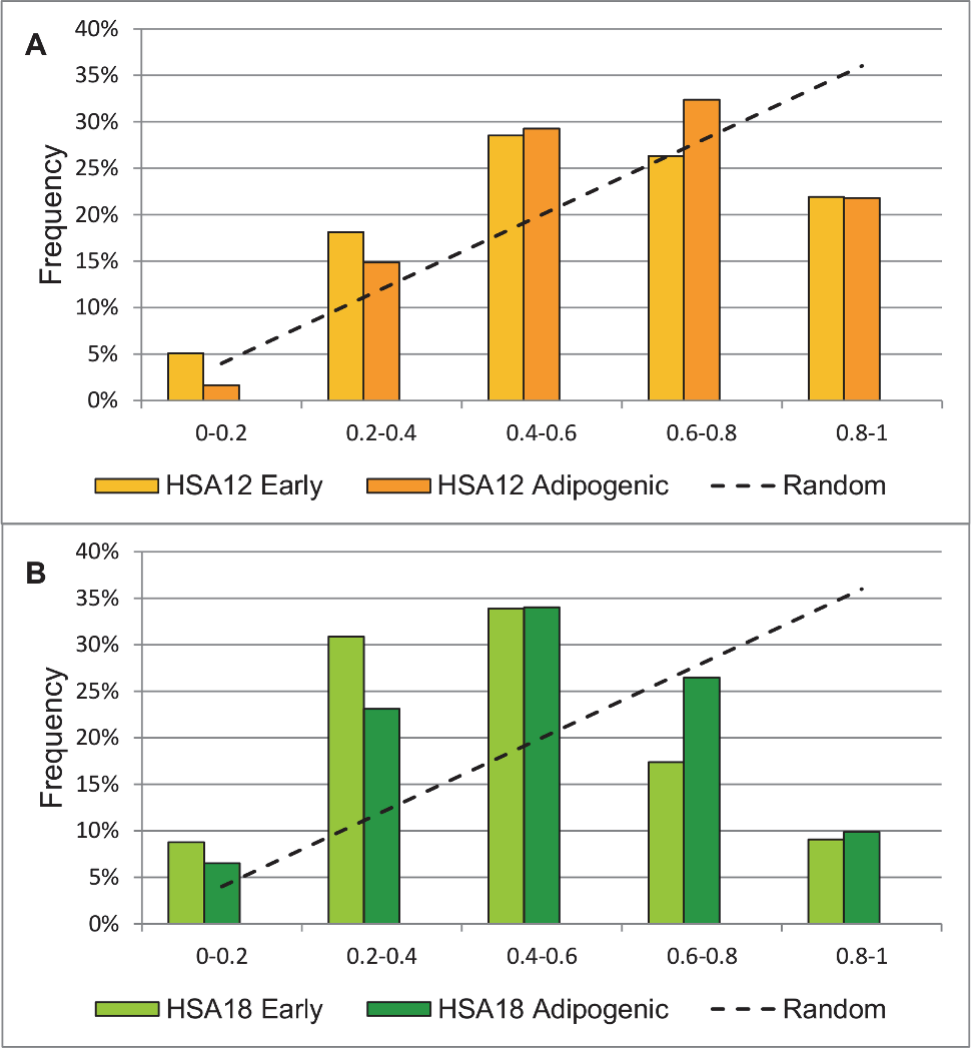
RDCs of HSA12 and HSA18 before and after adipogenic differentiation. RDCs of HSA12 (A) and HSA18 (B) both change after adipogenic differentiation of MSC (p = 0,008 for HSA12 and p = 0,0001 for HSA18). Dashed line demonstrates random distribution for comparison.

We compared RDCs of chromosomes before and after differentiation in adipogenic direction. Centromeres of both HSA12 and HSA18 are located more peripherally in differentiated cells (p = 0,008 for HSA12 and p = 0,0001 for HSA18). This changes are likely due to an increase of centromere location frequency in 0,6–0,8 interval compared to the higher frequency in 0–0,4 interval at early passages (Fig. 4). The distance between homologues centromeres of both chromosomes is also altered: two HSA12 homologues are found at a shorter distance after differentiation (0,0032±0,00009 and 0,0029±0,00009 before and after differentiation, respectively, p < 0,025, Kolmogorov-Smirnov Test). For HSA18 there is a small increase in the distance between homologues centromeres (0,0023±0,00005 and 0,0024±0,00008 before and after differentiation, respectively, p < 0,05, Kolmogorov-Smirnov Test). The analysis showed no significant difference in positions of HSA6 and HSAX centromeres before and after differentiation.

### Osteogenic differentiation changes position of HSAX centromere

**Fig 5:**
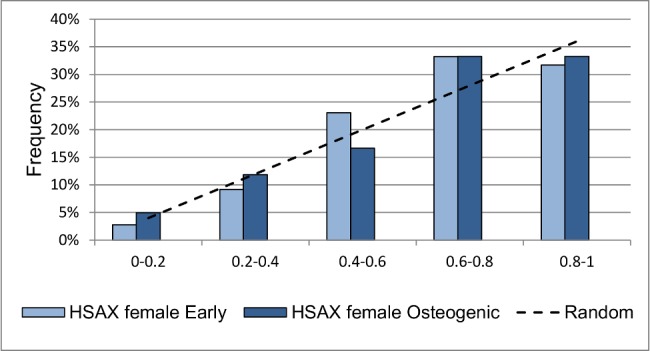
RDCs of HSAX in female cells before and after osteogenic differentiation. RDCs of HSAX in female cells change osteogenic differentiation of MSC (p = 0,04). Dashed line demonstrates random distribution for comparison.

HSAX centromere in female cells is differently distributed before and after osteogenic differentiation mostly in the 0–0,6 interval (p = 0,04) (Fig. 5). Though we did not detect differences in the distance between homologues, the angle between them increased after osteogenic differentiation (89,00±3,03 and 97,82±3,23, before and after differentiation, respectively, p <0,025). Distribution of HSAX centromere in male cells after differentiation differs from that in female cells (p = 0.04). We found no significant changes in positions of centromeres of HSA6, HSA12 and HSA18 before and after differentiation into osteogenic direction.

## Discussion

**Fig 6:**
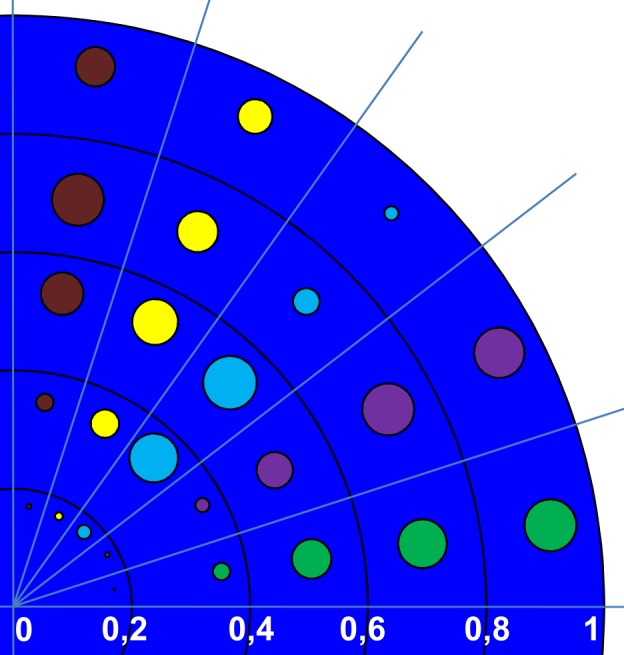
Schematic distribution of RDCs of HSA12, HSA18, HSA6 и HSAX in MSC nucleus. The sizes of the colored circles show the frequency of finding the centromere in the particular radial interval. Braun—HSA6, yellow—HSA12, light-blue—HSA18, violet—HSAX (female), green—HSAX (male).

We determined the positions of centromeres of HSA6, HSA12, HSA18 and HSAX in MSC nucleus. To our knowledge, this is the first time to report about positions of these chromosomes in human MSC at early and late passages and after differentiation. First we checked whether studied centromeres are placed in a non-random manner and found that RDCs frequency distributions of HSA6, HSA12 HSA18 significantly differ from the random distribution (Fig. 2) except HSAX in both male and female cells. One possible explanation of the failure to identify the non-random distribution of HSAX centromere is that it is really randomly localized in the nucleus, as it was shown for example for the HSAX in normal human blastomeres [26]. On the other hand this pattern may be explained by the superposition of the localization of chromosomes in different cell sub-populations, which existence is typical for MSCs. However from our point of view the most probable explanation is the insufficient number of analyzed cells to reach statistical significance. Indeed both ‘not significant’ graphs of HSAX centromere have much similarity with each other (males and females) and their shapes are closer to what we observe for HSA6 centromere than for random distribution. Indeed, after we merged the data for HSAX from male and female cells together (there is now difference between them, p = 0.57 and they are definitely similar to each other) we detected the expected difference from the random distribution (p = 0.03). We also found that centromeres of HSA12 and HSA6 consistent with previous reports are mainly localized at the distance of 60–80% of nuclear radius, which correlates to published data with using centromeric probes (HSA12) or whole chromosome probes (HSA6, HSA12) [9, 27]. HSA18 centromere takes the most proximal position of all chromosome centromeres we studied, which distinguishes MSCs from other previously studied cell cultures (ex. lymphocytes, [28]), where gene-poor HSA18 is usually located at the very periphery of the nucleus. The most distally located centromere is HSAX (Fig. 6).

We have shown that cellular processes such as differentiation in adipogenic and osteogenic directions and cultivation affect the positions of centromeres of different chromosomes. RDC of HSAX changes its after cultivation and after differentiation in osteogenic direction (Fig. 3 and 5) and chromosomes 12 and 18 change localization of their centromeres after adipogenic differentiation. It’s interesting to note different patterns of localization at the same conditions of HSAX centromeres in female and male cells. At early passages and after adipogenic differentiation HSAX centromere is distributed in the same manner both in male and female cells and in both cellular states. But after long cultivation and osteogenic differentiation HSAX centromere is differently distributed in female and male cells, changing it localization in female cells after differentiation and in male cells after long cultivation. The processes activated during differentiation and cultivation might be interconnected with each other, because it is known that cells at late passages prone to spontaneous differentiation. It’s also a well-known fact that active chromosomes tend to be localized in the nuclear center while silenced chromosomes are mainly localized at the nuclear periphery. And we may suggest that observed changes of localization of a certain centromere in the nucleus reflect the positional changes of the chromosome which in turn change levels of gene expression of these chromosomes as a result of ageing or/and differentiation.

**Fig 7:**
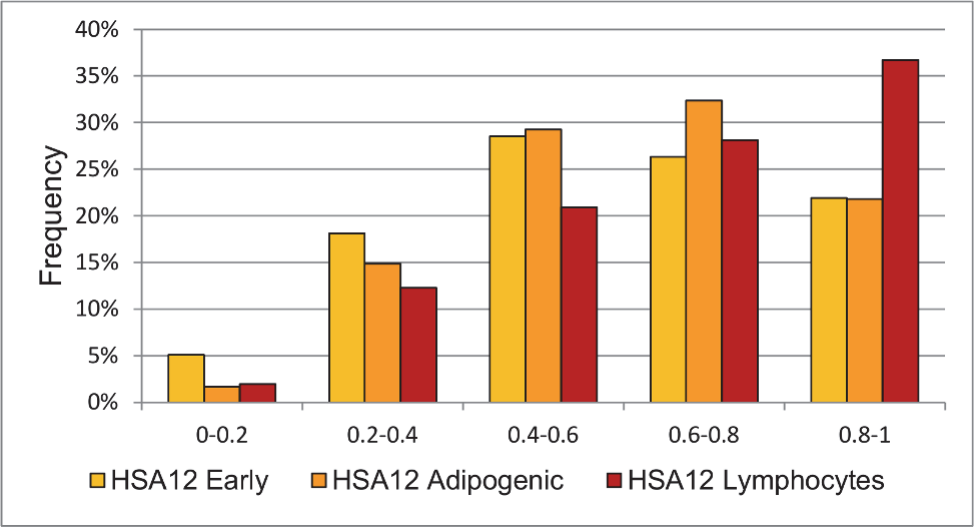
RDCs of HSA12 in MSC at early passages, after adipogenic differentiation and in lymphocytes. RDCs of HSA12 differ between MSC cells at early passages, after adipogenic differentiation (p = 0,008) and lymphocytes (p<10^–9^).

HSA12 centromere shows an attractive argument to confirm this speculation. According to the published data, in human embryonic stem cells (hESC) RDC of HSA12 is 56,2%±1,9% [9]. In human fibroblasts HSA12 is localized at 61,12%±19,52 (SD) [27]. We also studied human lymphocytes for HSA12 centromere localization and found it significantly more distal compared to MSC (59% and 71% in MSC and lymphocytes, respectively, p <10^–9^) (Fig. 7.) We conclude that HSA12 centromere occupies in MSCs more distal position compared to hESCs. In more differentiated cells like fibroblasts and lymphocytes position of HSA12 centromere becomes even more distal. According to the published data, the chromosome 12 contains a number of genes associated with the undifferentiated cellular status [29–31] as well as genes which expression is reduced in the process of differentiation [30, 32]. It is also known that 12p occupies in hESCs a proximal position as compared to differentiated cells like LCL [30]. Based on the published data and our results, it can be assumed that the more distal placement of HSA12 after differentiation might be associated with a reduced activity of its genes after differentiation.

## Conclusion

The importance of the chromosome position in the nucleus is supported by many evidences including evolutionary conservation of this trait and at the same time tissue specific patterns [8]. However we still lack the knowledge about specific changes of chromosome localization during cellular processes including differentiation and ageing and many stem cells including widely used MSC are poorly studied for the role of chromosome spatial rearrangements.

In this study we describe centromere positions of several chromosomes in MSC at early and late stages of cultivation as well as after *in vitro* differentiation to adipogenic and osteogenic directions. We demonstrate that as other cell types MSCs keep non-random cell-type specific pattern of chromosome positions. And these positions change during cultivation and differentiation.

## Supporting Information

S1 Dataset The file contains 5 Sheets: 1. “RDCs and Calc for Graphs” contains RDCs for each chromosome at every experimental condition (in columns B-Z) and for each cell analyzed (in lines). Summarized data about distribution of centromeres is available in cells AC1-BB8. Calculations of χ2 and data for Figures (Sheet “Graphs” see below) are in AC11-AW129; p-values are marked with bold border line. 2. “Distance” contains distances between two homologous centromeres. 3. “Angles” contains angles between two homologous centromeres. 4. “3D data” contains RDCs in 3D scanned nuclei for each chromosome at every experimental condition (in columns B-P) and for each cell analyzed (in lines). Summarized data about distribution of centromeres is available in cells T1-AT8. Calculations of χ2 are in T21-AT43; p-values are marked with bold border line. 5. “Graphs” contains Figures used in the article.
(XLSX)
